# Role of Vitamin D Replacement on Health Related Quality of Life in Hospitalized Patients with "Acute Exacerbation of Chronic Obstructive Pulmonary Disease"

**Published:** 2018

**Authors:** Mouhamad Hasan Pourrashid, Farzaneh Dastan, Jamshid Salamzadeh, Alireza Eslaminejad, Maryam Edalatifard

**Affiliations:** a *Department of Clinical Pharmacy, School of Pharmacy, Shahid Beheshti University of Medical Sciences, Tehran, Iran. *; b *Chronic Respiratory Disease Research Center, National Research Institute of Tuberculosis and Lung Disease (NRITLD), Shahid Beheshti University of Medical Sciences, Tehran, Iran.*; c *Department of Internal Medicine, School of Medicine, Tehran University of Medical Sciences, Tehran, Iran.*

**Keywords:** Chronic Obstructive Pulmonary Disease (COPD), Exacerbation, Vitamin D, Quality of Life, St. Georgeʹs Respiratory Questionnaire (SGRQ)

## Abstract

Burden of Chronic Obstructive Pulmonary Disease (COPD) is substantial and increasing in the world. There are controversial reports regarding vitamin D supplementation in COPD. We investigated relationship between vitamin D3 (Cholecalciferol) supplementation with Health Related Quality of Life (HRQOL) and symptom recovery in AECOPD patients with concurrent Vitamin D Deficiency (VDD). A placebo-controlled randomized clinical trial was designed. AECOPD patients with VDD were randomly allocated to receive either vitamin D 300,000 IU (n = 35) or placebo (n = 35) by intramuscular injection. Primary outcomes included the HRQOL assessed by St. Georgeʹs Respiratory Questionnaire (SGRQ), and the symptom recovery evaluated by the modified Medical Research Council (mMRC) Dyspnea scale, determined at baseline and at days 30 and 120 post-intervention. Secondary endpoints were length of hospital stay (LOS), rehospitalization and mortality rates.

Sixty-two patients, 30 patients in the vitamin D and 32 patients in the placebo groups, with mean ± SD age of 63.42 ± 8.48 years accomplished the study. Baseline vitamin D levels in the vitamin D and placebo groups were 10.59 ± 3.39 and 11.12 ± 3.17 ng/mL, respectively. These levels reached 36.85 ± 11.80 and 12.30 ± 3.66 in the vitamin D and placebo groups, respectively, at day 120 (*p < *0.001). Correction of vitamin D levels in the intervention group resulted in statistically significant improvement in patients’ HRQOL by day 120 compared to that of the placebo group (*p = *0.001); however no significant difference was observed in LOS, rehospitalization, and mortality rates. Single parenteral high dose of vitamin D as adjunctive therapy could improve HRQOL in hospitalized AECOPD patients with deficient levels of vitamin D.

## Introduction

Chronic Obstructive Pulmonary Disease (COPD) was known as the third leading cause of death in the world and had resulted in over 3.1 million deaths globally in 2012 (1). In addition, prevalence and burden of COPD is risen over time (2). Majority of this burden is attributed to hospitalization and disease exacerbations (3). COPD bares a substantial burden for patients, family, and the health care system (4). Patients with COPD had significantly worse activities of daily living and physical, social, and emotional functioning than the patients with non-small cell lung cancer (5) and Quality of life is significantly impaired in COPD patients (5-7), even in those with mild airway obstruction (8). Vitamin D Deficiency (VDD) as global health problem was shown to be a modifiable explanatory factor to improve outcomes in several chronic diseases (9). In a recent meta-analysis on COPD patients, high prevalence of VDD was reported, especially during disease exacerbation (10). VDD was associated with disease severity, poor quality of life, reduced lung function, longer hospital stay, frequent exacerbations, hospitalization, reduced physical functioning, poor balance and less muscle strength in COPD patients (10-13). Key role of vitamin D in inflammation process and COPD associate comorbidities including respiratory infection, cardiovascular disease, skeletal muscle dysfunction, depression and anxiety (14-18) as well as vitamin D role in enhancement of patients’ response to steroid therapy (19), open an interesting field to explore effects of the vitamin D on clinical outcomes such as HRQOL and dyspnea scale in AECOPD patients. 

Data regarding vitamin D supplementation in COPD is still controversial but promising (20). Although, some studies have shown no significant improvement in clinical outcomes such as dyspnea severity (21), physical performance (22), quality of life (23), and lung function of COPD patients receiving vitamin D supplement (22, 24), several studies revealed that vitamin D supplementation could improve lung function (25), reduce exacerbation rates (25, 26), improve in inspiratory muscle strength and maximal oxygen uptake (27), and also improve dyspnea scale, physical performance, maximum voluntary ventilation and maximal inspiratory pressure (28). In this study, we assessed effect of rapid normalization of vitamin D levels, using recommended high dose intramuscular vitamin D injection (29), on clinical outcomes including HRQOL, symptom recovery (dyspnea scale), length of hospital stay (LOS), mortality rate, and rehospitalization, in patients hospitalized with AECOPD.

## Experimental

*Study design and patients*

The study was designed as a randomized double-blind placebo-controlled trial, conducted at the Masih Daneshvari, a referral teaching hospital in Shahid Beheshti University of Medical Sciences, Tehran, Iran, from December 2015 to October 2016. The study was registered at the Iranian Clinical Trial Registry (IRCT 2016031425726N4) and approved by the University Ethics Committee (IR.SBMU.PHNM.1394.306). 

Estimated sample size for each study group was 28 patients, with α = 0.05 and power = 80%. Considering 20% dropout in the patients allocated in the study groups, total number of 70 patients (35 patients in each group) with COPD stages II-IV according to Global initiative for chronic Obstructive Lung Disease (GOLD) report 2015 (post-bronchodilator FEV1–FVC ratio 0.7, and had an FEV1 less than 80% predicted) entered the study. The participants were included if they admitted the diagnostic of AECOPD in the emergency ward and meet the inclusion criteria as below: Vitamin D deficiency (serum 25(OH)D < 20 ng/mL), age ≥ 40 years. Exclusion criteria included: serum 25(OH)D < 5 ng/mL, vitamin D supplementation within six month prior to study, use of maintenance dose of oral corticosteroids within three months prior to study, diagnosed asthma, osteoporosis, renal failure (serum createnine ≥ 2.5 mg/dL), nephrolithiasis, uncompensated liver failure (Child-pugh class B, C), hypercalcemia (ionized calcium > 1.3 mmol/L), conditions associated with pathological 1-alpha hydroxylase activity such as sarcoidosis, lymphoma or multiple myeloma, coagulopathy (platelet count < 30,000 per mm3 or international normalized ratio > 3), contraindication for intramuscular administration, pregnancy, lactation, and immuno-compromised patients *e.g.* patients on chemotherapy or transplant patients. Informed consent was obtained from all participants before their enrollment.

*Randomization and treatment design*

The permuted (balanced) block randomization was used for treatment allocation in this trial. The patients were randomly allocated to receive vitamin D3 (Cholecalciferol) 300,000 IU (Daropakhsh-Iran) or placebo as a single intramuscular injection. The patients in both groups were on standard treatment for AECOPD according to GOLD report 2015. Standard treatment included: [1] short-acting inhaled beta2-agonists with or without short-acting anticholinergics, [2] A dose of 40 mg prednisone per day for 5 days or nebulizer budesonide, [3] Antibiotics for patients with three cardinal symptoms (increase in dyspnea, sputum volume, and sputum purulence), or those who have two of the cardinal symptoms, if increased purulence of sputum is one of the two symptoms or require mechanical ventilation, for 5-10 days (18). 

*Study outcomes*

Primary outcomes included HRQOL of the patients, which was assessed by St. Georgeʹs Respiratory Questionnaire (SGRQ), and symptom recovery which was evaluated by the modified Medical Research Council (mMRC) dyspnea scale. Furthermore, the secondary end points were lengths of hospital stay (LOS), rehospitalization, and mortality rates during 120-day of study period.

*Measurements and follow-up*

Data regarding SGRQ and mMRC scores were collected at three different points in time: at baseline and follow up at day 30 and day 120 post-intervention. 

The SGRQ is the most comprehensive disease specific health related quality of life questionnaire (HRQOL) designed to measure and quantify health-related health status in patients with chronic airflow limitation (18). SGRQ evaluates symptoms, activities, and the impact of the disease on daily life of the patients. Scores range from 0 to 100 (maximum impairment) and decrease of at least four points in the total score of the SGRQ was defined as a clinically significant improvement in quality of life (30).

The modified Medical Research Council (mMRC) scale was used to assess the severity of patientʹs dyspnea. Dyspnea as cardinal symptom of COPD is a major cause of disability and anxiety associated with the COPD. mMRC Score ranges from 0 to 4, with higher scores, indicating more severe dyspnea (18).

Detailed data about medical history which were recommended by GOLD guideline including patient exposure to risk factors such as smoking, occupational or environmental exposure, past medical history, history of exacerbation and previous hospitalization for respiratory disorder, presence of comorbidity, demographic data, and relevant para-clinic or laboratory finding were also collected for further analysis.

*Statistical analysis*

Data analysis was conducted with IBM SPSS Statistics v 21.0 software. Continuous variables were assessed for normality by “Kolmogorov-Smirnov” test, and according to its results, *t*-test (normal distribution) or Mann–Whitney U-test (non-normal distribution) was used for between groupʹs comparisons. Continuous variables expressed as mean ± Standard Deviation (SD) or median (interquartile range) where appropriate. Categorical and nominal variables were expressed as frequency (%) and were analyzed using Chi-square test or Fisher’s-exact test. Repeated measure analysis was also used to compare vitamin D effect on patient HRQOL (SGRQ) and dyspnea severity (mMRC). Pearson or Kendellʹs Rank correlations were applied to assess the association between vitamin D at baseline and clinical finding. *P*-values < 0.05 were considered statistically significant.

## Results

*Patients’ characteristics*

**Figure 1:**
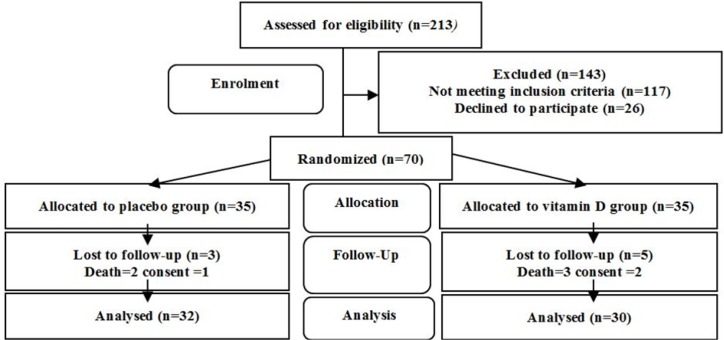
Study flowchart.

Out of 213 patients assessed for eligibility, 85 patients met the primary criteria. They were assessed for vitamin D level. Six patients with serum levels of 25(OH)D < 5 ng/mL and 9 patients with serum levels of 25(OH)D > 20 ng/mL were excluded from the study. Therefore, 70 patients with VDD were allocated equally in the study groups. Finally, 30 patients in the vitamin D group and 32 patients in the placebo group accomplished the clinical trial (Figure 1).

The baseline characteristics of the 62 patients included in the placebo and vitamin D groups are shown in Table 1. There was no statistically significant difference in baseline variables between vitamin D and placebo groups (*p *> 0.05).

*Change in vitamin D levels*

At baseline, mean ± SD of serum 25(OH)D levels were 10.59 ± 3.39 ng/mL and 11.12 ± 3.17 ng/mL in vitamin D and placebo groups respectively and did not differ in between groups comparison (*p = *0.82). Vitamin D supplementation resulted in a statistically significant increase in serum 25(OH)D levels in vitamin D group (36.85 ± 11.80 ng/mL) versus placebo group (12.30 ± 3.66 ng/mL), by day 120 [*p = *0.000, (CI -30.0, -18.90)].

*Primary outcomes*

*Changes in HRQOL (SGRQ)*

Baseline SGRQ scores included symptom, activity, impact and total scores were not statistically different in between group’s comparison (Table 2). Mean ± SD of the differences between baseline and the 30^th^ and 120^th^ days of SGRQ scores and its components are presented in Table 2.

Mean ± SD differences between baseline and day 30 of SGRQ total score were 3.73 ± 1.84 and 5.69 ± 2.13 units for placebo and vitamin D groups, respectively. Since mean difference of 1.96 in between groups, was less than 4 units, improvement in HRQOL in vitamin D group was not considered clinically significant, by day 30. However, between groups comparison of the differences of the SGRQ total scores at baseline and day 30, revealed a statistically significant variation [*p = *0.0003, (CI -2.96, -0.94)].

Mean ± SD differences between baseline and day 120 of SGRQ total score were 3.99 ± 2.19 and 8.66 ± 3.32 for the placebo and vitamin D groups, respectively. Since mean difference of 4.67 in between groups, exceeded the minimum clinically important difference of 4 units (30), the improvement in HRQOL in vitamin D group was considered clinically significant, by day 120. Between group comparison of the differences of the SGRQ total scores at baseline and day 120, showed a statistically significant difference between two groups [*p = *0.0001, (CI -6.11, -3.23)].

Repeated measures analysis of variance, showed statistically significant improvement in SGRQ total scores in vitamin D group compared with the placebo (F (1.74, 109.41) = 33.56, *p < *0.001). 

*Changes in Dyspnea Severity Scale (mMRC)*

**Figure 2:**
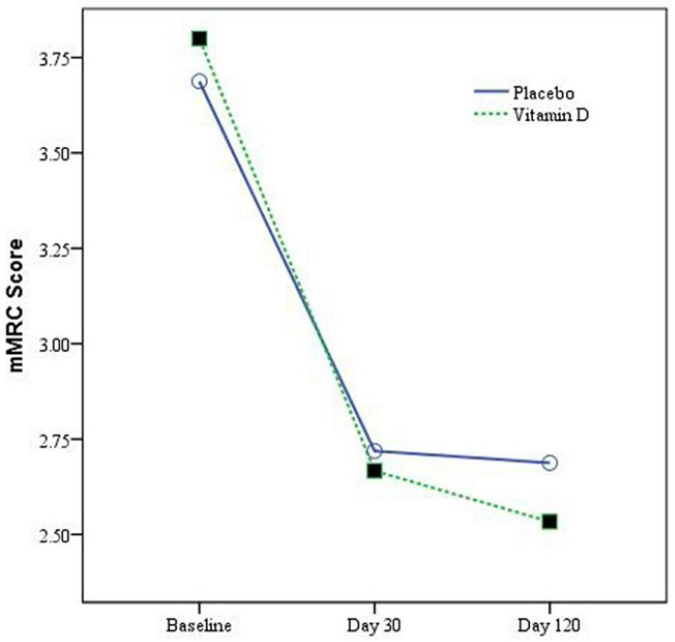
Change in mMRC score during study period in vitamin D and Placebo groups.

The mMRC scores at baseline in placebo and vitamin D groups were not statistically different (*p = *0.31) (Table 1). By day 30 change in mMRC scores were 0.97 ± 0.78 and 1.13 ± 0.82 in placebo and vitamin D groups respectively (*p = *0.44). By day 120, the change in mMRC scores were 1 ± 0.76 and 1.27 ± 0.78 in placebo and vitamin D groups respectively (*p = *0.17). There is no statistically significant difference in between group comparison. Change in mMRC score during study period in vitamin D and placebo groups was showed in Figure 2. 

Repeated measures analysis of variance, showed statistically non-significant changes in mMRC scores in vitamin D group compared to the placebo (F (2.05, 127.01) = 0.76, *p = *0.48). 

*Secondary outcomes*

The mean ± SD and [Median (interquartile range)] length of hospital stay was 8.06 ± 3.46, [7 (6.91, 9.20)], 7.37 ± 2.59, and [7 (6.53, 8.55)] days in placebo and vitamin D groups, respectively, which did not differ significantly in between group comparison [*p = *0.36]. Rehospitalization rates during the study period were 48.57% (n = 17) and 37.14% (n = 13) in the placebo and vitamin D groups, respectively (*p = *0.33). Mortality was reported in 2 and 3 patients in the placebo and vitamin D groups, respectively (*p = *1.00).

## Discussion

A randomized clinical trial (RCT) was designed to explore the effect of intramuscular high dose of vitamin D on HRQOL, in hospitalized AECOPD patients with coexisting vitamin D deficiency. This study was different in some aspects from previous trials. Firstly, most of the previous studies have focused on clinical outcomes such as exacerbation rate, readmission, exercise capacity, and mortality rate (22, 24-26 and 28) and also data about the effect of vitamin D on health related quality of life using a comprehensive questionnaire such as SGRQ are lacking. Secondly, in many of the previous researches, patients with sufficient levels of vitamin D were also included in the study (21, 23-25) Thirdly, in these studies, patients with stable disease were also recruited (22, 24-26) and trials on AECOPD population are lacking. Lastly, most of the previous studies have used frequently administered oral dosing of vitamin D (21, 23 and 25-28), in which, patient compliance might have affected their final findings. 

In our RCT, the prevalence of VDD among AECOPD patients was high (89.40%) at baseline. Vitamin D levels were well-correlated with disease severity and this was in accordance with recent meta-analysis in COPD patients reported in 2016 (10). Our study showed significant impaired quality of life in AECOPD patients, demonstrated by overall high SGRQ total scores, which was correlated with disease severity (r = 0.91, *p < *0.001). This was confirmed by previous reports (11). 

In vitamin D group, rapid normalization of vitamin D levels with a recommended high dose of vitamin D 300,000 IU (29) resulted in significant statistical improvement in HRQOL of the patients, by day 30 [*p = *0.0003, (CI -2.96, -0.94)] and day 120 [*p = *0.0001, (CI -6.11, -3.23)]; however, this improvement was not considered clinically significant by Day 30 and reached clinically significant level by day 120, since mean difference of 4.67 in between groups comparison exceeded the minimum clinically important difference of 4 units (30) . Improvement in other clinical end points such as mMRC (*p = *0.48), LOS (*p = *0.36), rehospitalization (*p = *0.33), and mortality rates (*p = *1.00) were not statistically significant. 

**Table 1:** Baseline characteristics of the patients

**Variable**	**Placebo group (n = 32)**	**Vitamin D group (n = 30)**	**p-value**
Age (year)	*64.06 ± 8.77	^*^62.73 ± 8.26	0.54
Gender	Male: 27 (84.38%) Female: 5 (15.62%)	Male: 25 (83.33%) Female: 5 (16.67%)	0.91
Education	No: 6 (18.75%) Elementary: 16 (50%) High school: 9 (28.13%) Academic: 1 (3.12%)	No: 5 (16.67%) Elementary: 17 (56.67%) High school: 6 (20%) Academic: 2 (6, 67%)	0.80
Marital status	Unmarried: 2 (6.25%) Married: 24 (75%) Divorced, widowed: 6 (18.75%)	Unmarried: 0 (0%) Married: 25 (83.33%) Divorced, widowed: 5 (16.67%)	0.36
Income	Low: 6 (18.75%) Middle: 17 (53.13%) Upper middle: 4 (12.5%) High: 3 (9.38%)	Low: 6 (20%) Middle: 13 (43.33%) Upper middle: 6 (30%) High: 2 (6.67%)	0.81
BMI (kg/m²)	^*^22.90 ± 1.97	^*^22.99 ± 1.69	0.85
Smocking (pack/year)	**50 (40, 58)	**50 (40, 50)	0.42
Water pipe use	11 (34.38%)	12 (40%)	0.65
Smoking status	Ex-smokers: 25 (78.13%) Current smokers: 7 (21.88%)	Ex-smokers: 23 (76.66%) Current smokers: 7 (23.33%)	0.89
COPD Stage	II: 16 (50%)III: 11 (34.38%)IV: 5 (15.63%)	II: 13 (40.63%)III: 12 (40%)IV: 5 (16.67%)	0.87
AECOPD Severity	Moderate: 10 (31.25%) Severe: 22 (68.75%)	Moderate: 10 (33.33%) Severe: 20 (66.67%)	0.86
Exacerbation History	< 3 times/year: 17 (53.13%) ≥ 3 times/year: 15 (46.87%)	< 3 times/year: 14 (46.67%) ≥ 3 times/year: 16 (53.33%)	0.61
Presence of co-morbidities	Hypertension: 14 (43.75%)	Hypertension: 8 (26.67%)	0.16
	Diabetes: 2 (6.25%)	Diabetes: 3 (10%)	0.59
	Depression/Anxiety: 9 (18.75%)	Depression/Anxiety: 6 (20%)	0.46
	IHD: 4 (12.50%)	IHD: 5 (16.67%)	0.64
	Stroke: 2 (6.25%)	Stroke: 1 (3.33%)	0.59
	HF: 4 (12.5%)	HF: 3 (10.00%)	0.93
Drug	LABA: 7 (21.88%)	LABA: 4 (13.33%)	0.38
	ICS: 9 (28.13%)	ICS: 8 (26.67%)	0.46
	LABA+ICS: 22 (68.75%)	LABA+ICS: 24 (80%)	0.31
	LAMA: 16 (50%)	LAMA: 17 (56.67%)	0.60
	Theophylline: 5 (15.63%)	Theophylline: 4 (13.33%)	0.80
	Acetylcysteine: 8 (25%)	Acetylcysteine: 9 (30%)	0.66
	Statin: 8 (25%)	Statin: 5 (16.67%)	0.42
Morisky adherence score	**5 (4,6)	**5 (4,6)	0.86
Vitamin D (ng/mL)	*11.01 ± 2.99	*10.82 ± 3.73	0.82
WBC cont × 10³ (cell/μL)	8.33 ± 2.23	8.66 ± 2.69	0.62
Calcium (mg/dL)	**9.1 (8.65, 9.40)	**9.2 (8.75, 9.45)	0.89
Phosphor (mg/dL)	**3.2 (2.95, 3.45)	**2.7 (3.1, 3.75)	0.39
SGRQ (Symptom)	*81.68 ± 8.78	*85.06 ± 8.37	0.13
SGRQ (Activity)	**78.96 (63.97, 85.81)	**85.66 (71.20, 87.16)	0.13
SGRQ (Impact)	*54.15 ± 15.33	*55.98 ± 14.12	0.63
SGRQ (Total)	*64.97 ± 12.28	*67.99 ± 10.67	0.31
Dyspnea scale (mMRC)	Grade 3: 10 (31.25%)Grade 4: 22 (68.75%)	Grade 3: 6 (20%)Grade 4: 24 (80%)	0.31

* Data has been presented as mean ± SD.

** Data has been presented as median (interquartile range 25^th^, 75^th^ percentiles), BMI: Body Mass Index, IHD: Ischemic Heart Disease, HF: Heart Failure LABA: Long Acting Beta 2 Agonist, LAMA: Long-Acting Muscarinic Antagonist, ICS: Inhaled Cortico-Steroid, SGRQ: ST George’s Respiratory Questionnaire, mMRC: modified Medical Research Council.

**Table 2:** Differences between baseline and the 30^th^ and 120^th^ days of SGRQ scores and its components in the study groups.

**SGRQ component**	**By day 30**			**By day120**		
	**Placebo**	**Vitamin D**	**p-value**	**Placebo**	**Vitamin D**	**p-value**
Symptom score	10.71 ± 6.44	15.18 ± 8.85	0.050	12.26 ± 7.81	19.45 ± 8.94	0.001*
Activity score	1.88 ± 3.73	4.11 ± 3.43	0.008*	2.69 ± 3.96	8.11 ± 6.13	0.001*
Impact score	2.61 ± 3.20	3.77 ± 3.82	0.40	2.13 ± 4.39	5.76 ± 4.62	0.007*
Total score	3.73 ± 1.84	5.69 ± 2.13	0.0003*	3.99 ± 2.19	8.66 ± 3.32	0.0001*

* Significant improvement in vitamin D group versus placebo (p < 0.05)

The data about vitamin D supplementation in COPD patients have resulted in controversial but promising findings (20). Some studies have reported no beneficial effect for vitamin D on exercise capacity of the patients including 6-minute walking test, saturation of oxygen during exercise, COPD assessment test score, and pulmonary function test (22, 24). On the other hand, beneficial effect of vitamin D supplementation has been documented in several studies. In a study by Lehouk *et al*. vitamin D supplementation in 30 participants with coexisting VDD showed a significant reduction in exacerbations; however, pulmonary function, hospitalization and mortality did not significantly differ between study groups (31). In a study by Rezk *et al*. carried out on COPD patients with VDD, a significant improvement in dyspnea scale (mMRC), physical performance, maximum voluntary ventilation, maximum inspiratory pressure, and maximum expiratory pressure, coupled with a decrease in disease exacerbations and CRP a year after vitamin D replacement were reported. However, the FEV1 and FVC did not differ significantly (28). In another study by Martineau *et al.* vitamin D was protective against moderate or severe exacerbations in COPD patients with VDD (26). In an RCT by Zendedel *et al*. vitamin D supplementation resulted in significant improvement in FEV1 and significant decrease in the number of COPD exacerbations of COPD patients (25). It is also shown that vitamin D supplementation can result in significantly greater improvements in inspiratory muscle strength and maximal oxygen uptake. However, improvements in quadriceps strength or six minutes walking distance test could not reach a significantly improved level (27).

Based on our literature review, the data about the effect of vitamin D on the quality of life of the COPD patients, determined by a comprehensive tool such as SGRQ, were limited to one study by Bjerk *et al. *It was performed on Caucasian male subjects. The results revealed no improvements in HRQOL (SGRQ) and physical activity of the study patients. This study has several limitations such as small sample size (n = 36) and a short follow up period (6 weeks). The differences between this and our study were vitamin D dosing (multiple low oral dosing versus single high intramuscular dosing of vitamin D), study subjects (stable COPD versus AECOPD patients), and inclusion criteria (which was not included vitamin D deficiency) (23).

 In a recent study on asthmatic patients by Rajanandh *et al*. vitamin D supplementation (1000 IU/day) resulted in statistical as well as clinical improvement in all domains of the SGRQ including symptom score, impact score, activity (from day 30 onward), and total score by day 180 (32). The results were in agreement with our study by the difference in the study populations.

In the interpretation of the results obtained in our study and their comparison with those described in the previous reports, methodological and clinical differences including dose and route of the vitamin D administration, disease related factors, genetic features of the study population, inclusion and exclusion criteria and characteristics of the patients should be acknowledged.

*Limitations of the study*

Patients included in this study were AECOPD patients with concomitant VDD; therefore, our findings may not be generalized to all AECOPD patients. A relatively short follow up period of 120 days was considered in this study, which may not have been long enough to document the delayed clinical outcomes resulted by the intervention.

## Conclusion

HRQOL in hospitalized AECOPD patients with concomitant deficient levels of vitamin D was significantly impaired. Vitamin D replacement with a single parenteral injection could be an effective intervention to improve patients’ HRQOL; however improvement in other clinical outcomes such as LOS, rehospitalization and mortality rates were not achieved. Further studies with a longer follow up period are recommended.
